# Mitochondrial Sirtuin 4 Resolves Immune Tolerance in Monocytes by Rebalancing Glycolysis and Glucose Oxidation Homeostasis

**DOI:** 10.3389/fimmu.2018.00419

**Published:** 2018-03-09

**Authors:** Jie Tao, Jingpu Zhang, Yun Ling, Charles E. McCall, Tie Fu Liu

**Affiliations:** ^1^Scientific Research Center, Shanghai Public Health Clinical Center, Fudan University, Shanghai, China; ^2^Molecular Medicine Section, Department of Internal Medicine, Wake Forest University, Winston-Salem, NC, United States

**Keywords:** monocytes/macrophages, acute inflammatory resolution, glucose metabolic homeostasis, sirtuin 4, pyruvate dehydrogenase complex, pyruvate dehydrogenase kinase

## Abstract

The goal of this investigation was to define the molecular mechanism underlying physiologic conversion of immune tolerance to resolution of the acute inflammatory response, which is unknown. An example of this knowledge gap and its clinical importance is the broad-based energy deficit and immunometabolic paralysis in blood monocytes from non-survivors of human and mouse sepsis that precludes sepsis resolution. This immunometabolic dysregulation is biomarked by *ex vivo* endotoxin tolerance to increased glycolysis and TNF-α expression. To investigate how tolerance switches to resolution, we adapted our previously documented models associated with acute inflammatory, immune, and metabolic reprogramming that induces endotoxin tolerance as a model of sepsis in human monocytes. We report here that mitochondrial sirtuin 4 (SIRT4) physiologically breaks tolerance and resolves acute inflammation in human monocytes by coordinately reprogramming of metabolism and bioenergetics. We find that increased SIRT4 mRNA and protein expression during immune tolerance counters the increase in pyruvate dehydrogenase kinase 1 (PDK1) and SIRT1 that promote tolerance by switching glucose-dependent support of immune resistance to fatty acid oxidation support of immune tolerance. By decreasing PDK1, pyruvate dehydrogenase complex reactivation rebalances mitochondrial respiration, and by decreasing SIRT1, SIRT4 represses fatty acid oxidation. The precise mechanism for the mitochondrial SIRT4 nuclear feedback is unclear. Our findings are consistent with a new concept in which mitochondrial SIRT4 directs the axis that controls anabolic and catabolic energy sources.

## Introduction

When stressed through TLR4 and other inflammatory and innate immune receptors, monocytes sequentially reprogram their highly conserved life-protection sequence of *resistance, tolerance, and resolution* (1). Emerging data support that metabolism and bioenergetics coordinately reprogram immune resistance and tolerance during acute inflammation, and that this integrated immunometabolic network dysregulates during many acute and chronic inflammation disorders (2, 3). Different metabolic and energy needs drive functions of distinct immune cell lineages, such as monocytes, macrophages, dendritic cells, T effector, and repressor cells (4), but the energy sources are consistent: glucose and amino acids support the metabolic and anabolic biosynthetic needs of the effector immune cells that resist inflection; fatty acid oxidation (FAO) with decreased lipogenesis support the catabolic processes associated with tolerance and immune repressor cells (5–7). Despite these gains in understanding how immunity and metabolism are integrated during resistance and tolerance, the reprogramming processes that promote inflammation resolution are unclear. The goal of this investigation was to identify the physiologic path that resolves the acute inflammatory response.

In this context, we have previously reported that epigenetic reprogramming of tolerance in human and mouse monocytes is directed by a nuclear sirtuin 1 (SIRT1) and SIRT6 axis (8) and SIRT1-RelB-SIRT3 axis (9). This NAD redox sensing checkpoint switches the glycolysis-dependent resistance phenotype to the FAO-dependent tolerance phenotype (10, 11). We also discovered that mitochondrial pyruvate dehydrogenase kinase 1 (PDK1) persistently deactivates pyruvate dehydrogenase complex (PDC), which is downstream of nuclear epigenetic reprogramming of tolerance by nuclear SIRT1 in mice and human septic monocytes. PDC is the rate-limiting step in decarboxylating pyruvate to acetyl CoA and oxidizing Krebs cycle glucose-based carbons that support oxidative phosphorylation (12). SIRT1 is a key regulator of fatty acid lipolysis and FAO (13). Glucose is known to primarily support anabolism and FAO to support catabolic processes. Dominance of catabolic energy over anabolic energy during tolerance deprives immune cells of glucose carbons needed to resist infection.

In this study of inflammation resolution, we adapted our published models of mouse and human acute inflammation that simulate sepsis, which use primary monocytes and THP-1-transformed monocytes to reprogram glucose anabolic fueling of the immune resistance phenotype to FAO fueling of the immune tolerance (10). Based on the known ability of mitochondrial sirtuin 4 (SIRT4) (14) to oppose the catabolic effects of mitochondrial SIRT3 that support fatty acid β oxidation and tolerance (15, 16), we surmised that SIRT4 might reprogram tolerance to resolution. Here, we report that SIRT4 breaks tolerance and promotes acute inflammation resolution in monocytes.

## Materials and Methods

### Chemicals and Standards

Bacterial endotoxin [lipopolysaccharide endotoxin (LPS)] was purchased from Sigma (*Escherichia coli* 0111:B4); d-[5-^3^H(N)]-glucose, d-[6-^14^C]-glucose, 1-[^14^C]-palmitic acid and hyamine were obtained from Perkin-Elmer; Mito stress kit was from Seahorse Bioscience; Cell-Tak was from BD company; colorimetric assay kits for pyruvate dehydrogenase (PDH), lactate dehydrogenase (LDH) activities and for pyruvate and lactate levels were from Biovision; gene-specific TaqMan primer/probe sets were from Applied Biosystems; SIRT4 gene-specific siRNA and control siRNA were from Santa Cruz Biotechnology; SIRT4 Flag (Plasmid #13815) (17), control pcDNA3.1+ without SIRT4 sequence, lentiCRISPRv2 vector, lentivirus helper plasmids psPAX2 and pND2.G were from Addgene; gene-specific antibodies were purchased from Gentex company.

### Modeling the Acute Inflammatory Response in THP-1 Human Monocytes

THP-1 cells from the American Type Culture Collection were maintained in RPMI 1640 medium (Invitrogen) supplemented with 100 U/ml penicillin, 100 µg/ml streptomycin, 2 mM l-glutamine, and 10% fetal bovine serum (HyClone, Logan, UT) in a humidified incubator with 5% CO_2_ at 37°C. Cells were stimulated with 1 µg/ml of bacterial endotoxin LPS (*Escherichia coli* 0111:B4, Sigma) for indicated times to generate inflammatory phases of initiation (0–8 h), endotoxin tolerance (24–48 h), and resolution (48–96 h). Endotoxin tolerance is determined after a second stimulation with LPS at 1 µg/ml for 1 h.

### Modeling the Acute Inflammatory Response in Human Peripheral Blood Mononuclear Cells

Healthy human blood samples were collected according to the protocol approved by Shanghai Public Health Clinical Center Ethics Committee, Fudan University. Peripheral blood mononuclear cells were purified by ficoll-hypaque density gradient centrifugation. Cells were maintained in complete RPMI 1640 culture medium for the indicated times in the presence or absence of 100 ng/ml LPS for SIRT4 gene expression and protein assay.

### Measuring Glucose and FAO

Oxidation of glucose and fatty acid was measured by radiolabeling as described previously (10). Briefly, cells were starved in triplicate in polypropylene vials for 30 min at 37°C in glucose-free Hank’s buffer. The vials were then placed into glass scintillation tube. After addition to cells 1 μCi of d-[6-^14^C]-glucose and 2.5 mM cold glucose or 1 μCi of 1-[^14^C]-palmitic acid in 0.2% BSA-Hank’s buffer, the scintillation tubes were sealed and rotated at 37°C in a water bath for 1 h. Metabolic reaction was then stopped by injecting 200 µl of 2N HCl into the central vials, and 500 µl of hyamine (Perkin-Elmer) was injected into the scintillation tube. ^14^CO_2_ generated by the oxidation of d-[6-^14^C]-glucose or 1-[^14^C]-palmitic acid was collected by overnight shaking at room temperature, ^14^CO_2_ in hyamine was counted using a β-counter. One μCi of d-[6-^14^C]-glucose alone or 1-[^14^C]-palmitic acid alone in the same amount of buffer was set for background counts.

### Glycolysis Assays

Glycolysis was measured by the conversion of d-[5-^3^H(N)]-glucose to tritiated water as described elsewhere (10). In brief, after starvation with glucose-free RPMI (Gibco) medium, Cells were incubated in triplicates with 1 μCi of d-[5-^3^H(N)]-glucose (Perkin-Elmer) at 37°C for 1 h in scintillation tubes containing 1 ml of H_2_O. The reaction was stopped by adding HCl (1N final) and scintillation tube was sealed. [^3^H]_2_O generated by enolase activity from d-[5-^3^H(N)]-glucose was vaporized for overnight at 50°C oven and cooled down for another overnight at 4°C. After removal of the central wells, [^3^H]_2_O was counted using a β-counter for detection of glycolytic rate. One μCi of d-[5-^3^H(N)]-glucose alone in triplicates was set for background control. One μCi of [^3^H]_2_O (Perkin-Elmer) alone in triplicates was set for detecting the efficiency of this water vapor exchange.

### Mitochondrial Respiration Monitoring

Mitochondrial oxidative phosphorylation reactions were assessed by comparing the ratio of oxygen consumption rate (OCR) and extracellular acidification rate (ECAR) with a cell Mito stress kit using an XFe-24 Extracellular Flux Analyzer (Seahorse Bioscience) (9). XFe sensor was pre-calibrated in XFe calibrate medium for overnight at 37°C in a CO_2_-free incubator. The 24-well microplate was pretreated for 20 min with Cell-Tak 3.5 µg/cm^2^ surface area (BD Biosciences) in 0.1 M sodium bicarbonate (pH 8.0) buffer. Excess Cell-Tak was removed by two washes with sterile water. THP-1 cells (2 × 10^5^/well) in bicarbonate-free and Hepes-free RPMI medium (pH 7.4, Invitrogen) supplemented with 2% FBS were set into a Cell-Tak pretreated microplate and pre-incubated for 1 h at 37°C in a CO_2_-free incubator. Then 10-fold concentrated compounds (Seahorse Biosciences) of oligomycin (Complex V inhibitor), carbonyl cyanide-*p*-trifluoromethoxyphenylhydrazone (FCCP, electron transport chain uncoupler), or a mixture of rotenone (Complex I inhibitor) and antimycin A (Complex III inhibitor) were loaded into a sensor cartridge to produce final concentration of them at 1 µM, 1.5 µM, 100 nM, and 1 µM, respectively. After a 30 min calibration of the XFe sensor with the pre-incubated sensor cartridge, the cell plate was loaded into the analyzer, OCR and ECAR were analyzed under basal condition and followed by sequential injection of the complex inhibitors oligomycin, FCCP, and the mixture of rotenone and antimycin A. Data were analyzed using XFe software (Seahorse Bioscience) and normalized with protein loaded in each well. Four replicates of each sample were analyzed.

### Enzyme Activity Assays

Colorimetric assay kits (Biovision) were used to biochemically assess the enzymatic activities of PDH and LDH according to the manufacture’s instruction. In brief, cells (1 × 10^6^) were lysed for 10 min in ice cold assay buffer, supernatants were collected by centrifugation and 10 µl of supernatant of each sample were loaded into 96-well plate in duplicates and adjusted the volume to 50 μl/well with assay buffer. Optic density (OD) at 450 nm was read immediately (OD-0) and 30 min (OD-30) after incubation at room temperature. Sample OD was calculated by subtracting OD-0 from OD-30. NADH standard curve was generated by adding 0, 2, 4, 6, 8, and 10 µl of 1.25 mM NADH Standard into a series of wells to generate 0, 2.5, 5.0, 7.5, 10, and 12.5 nmol/well of NADH Standard after adjusting the volume to 50 μl/well with assay buffer. Data were normalized with loaded protein.

### Intracellular Pyruvate and Lactate Levels

Intracellular pyruvate and lactate were measured by colorimetric assay kits (BioAssay Systems, CA, USA) according to manufacturer’s instruction. Briefly, 10 µl of samples or standards were transferred into 96-well plates in duplicates. Ninety microliters of working solution were added into each wells and incubated for 30 min at room temperature. Optical density at 570 nm was read and concentration of pyruvate or lactate was calculated from standard curve.

### Real-time RT-PCR

Levels of human TNF-α, SIRT1, SIRT4, and PDK1 mRNA were measured as describe previously (8–10) by quantitative real-time RT-PCR using gene-specific TaqMan primer/probe sets in an ABI prism 7000 Sequence Detection System (Applied Biosystems). GAPDH mRNA transcription was used for internal loading control.

### SIRT4 Gene-Specific Knockdown and Knockout

For SIRT4 knockdown, 60 pMol of a pool of three target-specific siRNA (Santa Cruz Biotechnology) were electronically transfected into responsive THP-1 cells for 24 h using Amaxa Nucleofector kit V and Amaxa nucleofector II device (Lonza, Inc.) and a pool of scrambled siRNAs was transfected as a negative control. The inhibition efficiency of knockdown is ~70% mRNA reduction on basal and after LPS stimulation.

For over-expression of SIRT4, THP-1 cells were electronically transfected with SIRT4 Flag (Plasmid #13815) (17) or pcDNA3.1+ without SIRT4 sequence for 24 h, cells were then processed for experiments. Basal SIRT4 mRNA level of SIRT4 plasmid transfected cells was 2.5-fold higher than that of control cells.

For SIRT4 knockout using CRISPR-Cas9 technique, the gRNA in lentiCRISPRv2 vector (Addgene, Cambridge, MA, USA) was designed as follows Oligo 1: 5′-CACCGGAGGCTCCTAGTAATGACCG-3′, Oligo 2: 3′-AAACCGGTCATTACTAGGAGCCTCC-5′. The lentiCRISPRv2 plasmid harboring the gRNA sequence and Cas9 gene, lentivirus helper plasmids psPAX2 and pND2.G (Addgene, Cambridge, MA, USA) were co-transfected into HEK-293T cells (American Type Culture Collection) using HG-trans293 (Healthgene, Canada) according to the manufacturer’s protocol. Virus-containing supernatants were collected 48 h and 72 h after transfection, respectively, filtered to eliminate cells, and infect target cells in the presence of 8 µg/ml polybrene. 72 h later, infected cells were cultured with 1 µg/ml puromycin for 7 days, puromycin resistant cells were set for experiments. LentiCRISPRv2 vector without SIRT4 gRNA sequence was used as control. SIRT4 gRNA containing cells reduced more than 90% SIRT4 protein level based on immunoblotting assay.

### Western Blot Analysis

Protein levels were analyzed as described previously (8). In brief, equal amounts (50 µg) of cell lysates were separated by SDS-PAGE electrophoresis and transferred to a polyvinylidene difluoride membrane (Perkin-Elmer Life Sciences). Blots were blocked with 5% milk-PBST for 1 h at room temperature and probed for overnight at 4°C using primary antibodies against SIRT4, PDK1, PDC, p-PDC E1α-S_232_ (Genetex), and GAPDH. Protein complexes were detected by incubation for 1 h at room temperature with secondary antibodies conjugated to horseradish peroxidase (Sigma) diluted at 1:2,000 in blocking buffer and then detected by Enhanced Chemiluminescence Plus (GE Healthcare). Protein binds were scanned and densitometry analysis was performed using ImageJ software.

### Statistical Analyses

Differences of immune metabolic changes between two related conditions were analyzed by Student’s *t*-test using GraphPad Prism version 6 (San Diego, CA, USA). *P*-values of less than 0.05 were considered significant. For experiments with more than two groups, Prism’s Two Way Analysis of Variance (ANOVA) was used with *post hoc* analysis of across multiple means. Exact *P* values are shown. Data points are Mean ± SEM of replicates.

## Results

### Simultaneous Immune Metabolic Switches Reprogram Physiological Resolution of Acute Inflammatory Response

**Figure 1:**
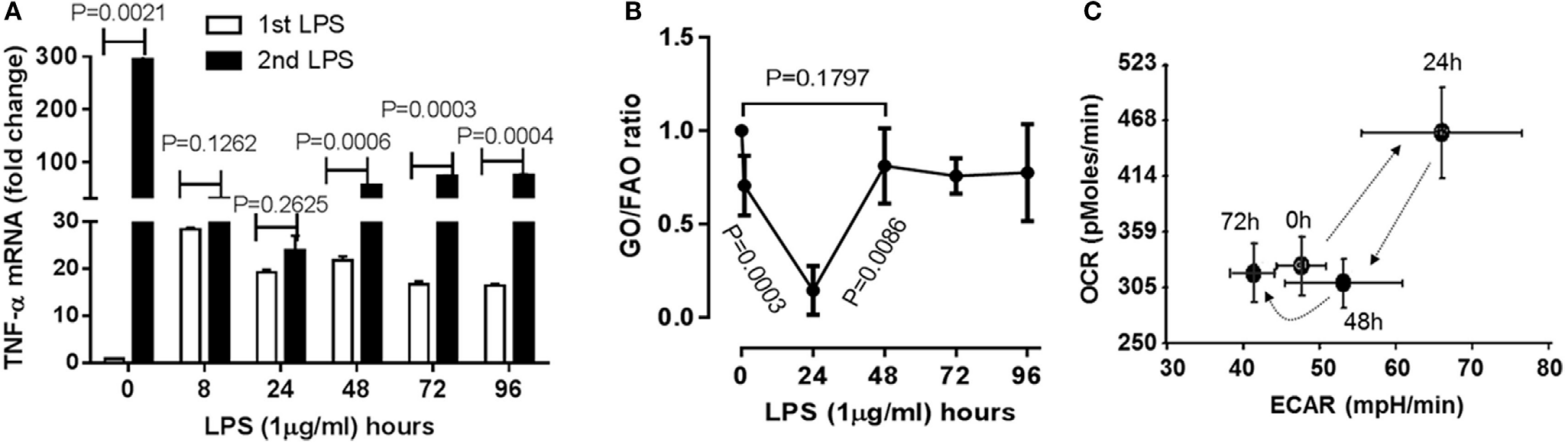
Time-dependent immunometabolic reprogramming of the acute inflammatory response to endotoxin in THP-1 human monocytes. **(A)** THP-1 cells received a primary dose of 1 µg/ml of lipopolysaccharide endotoxin (LPS) and were monitored for 96 h. A second LPS dose was given to track development and resolution of immune tolerance, as assessed by TNF-α mRNA induction by real-time RT-PCR. **(B)** Cellular metabolic rates of glucose (GO) and fatty acid oxidation (FAO) were analyzed using d-[6-^14^C]glucose and 1-[^14^C]palmitic acid, and expressed as GO/FAO ratios of ^14^CO_2_ production. **(C)** Oxygen Consumption Rate (OCR) and Extracellular Acidification Rate (ECAR) were determined using Seahorse XFe-24 Analyzer and energy index was platted as the ratio of the two. **(A)** *N* = 3, **(B)** *N* = 3, **(C)** *N* = 4. *P* values from analysis of variance and *post hoc* comparisons.

To understand if the physiological switch of immune suppression to inflammatory resolution also aligns with metabolic and energetic changes, we first timed the recovery of acute inflammatory response using a validated human monocyte THP-1 cell model of sepsis (8–10). THP-1 cells were stimulated with high dose (1 µg/ml) LPS for different times till 96 h. Pro-inflammatory cytokine TNF-α gene was monitored by real-time RT-PCR analysis. TNF-α mRNA of THP-1 cells was emerged peak transcription at 1 h (*P* = 0.0021), repressed at 8 h (*P* = 0.0025) and sustained at low level till 96 h after primary LPS stimulation. To determine the time of physiological recovery from immune suppression, THP-1 cells received second LPS stimulation at different time points after the first LPS stimulation. TNF-α transcription was not significantly induced at 8 h (*P* = 0.1262) and 24 h (*P* = 0.2625), indicating the generation of immune tolerance; however, TNF-α was significantly induced at 48 h after first LPS (*P* = 0.0006) and was gradually increased at 72 h (*P* = 0.0003) and 96 h (*P* = 0.0006) in response to second LPS stimulation (Figure 1A). This observation indicates that immune tolerant monocytes are physiologically able to resolve their immune repressor state.

As the immune tolerance in monocytes requires a switch from glucose to fatty acid metabolism (10), we reasoned the second switch from tolerance to resolution might reverse this alignment. To test this possibility, we assessed the dynamics of metabolic ratio of glucose oxidation (GO) to fatty acid oxidation (FAO) during the course of acute inflammatory response using radiolabeling analysis. Consistence with our premise, the ratio of ^14^CO_2_ production of ^14^C-glucose to that of ^14^C-palmitic acid was significantly decreased at 24 h (*P* = 0.0003), but increased 48 h after LPS stimulation (resolving switch, *P* = 0.0086) and restored toward the basal level (*P* = 0.1797, Figure 1B).

Concordant with this metabolic shift, the ratio of mitochondrial OCR and glycolytic ECAR increased at 24 h and returned to baseline at 48 h (Figure 1C) as described previously (9). These findings are consistent with the notion that physiological resolution of acute inflammation restores metabolic and energy homeostasis.

### Increases in SIRT4 Expression Parallel Development of Immune Tolerance Reprogramming and Precede Resolution of the Acute Inflammatory Response

**Figure 2:**
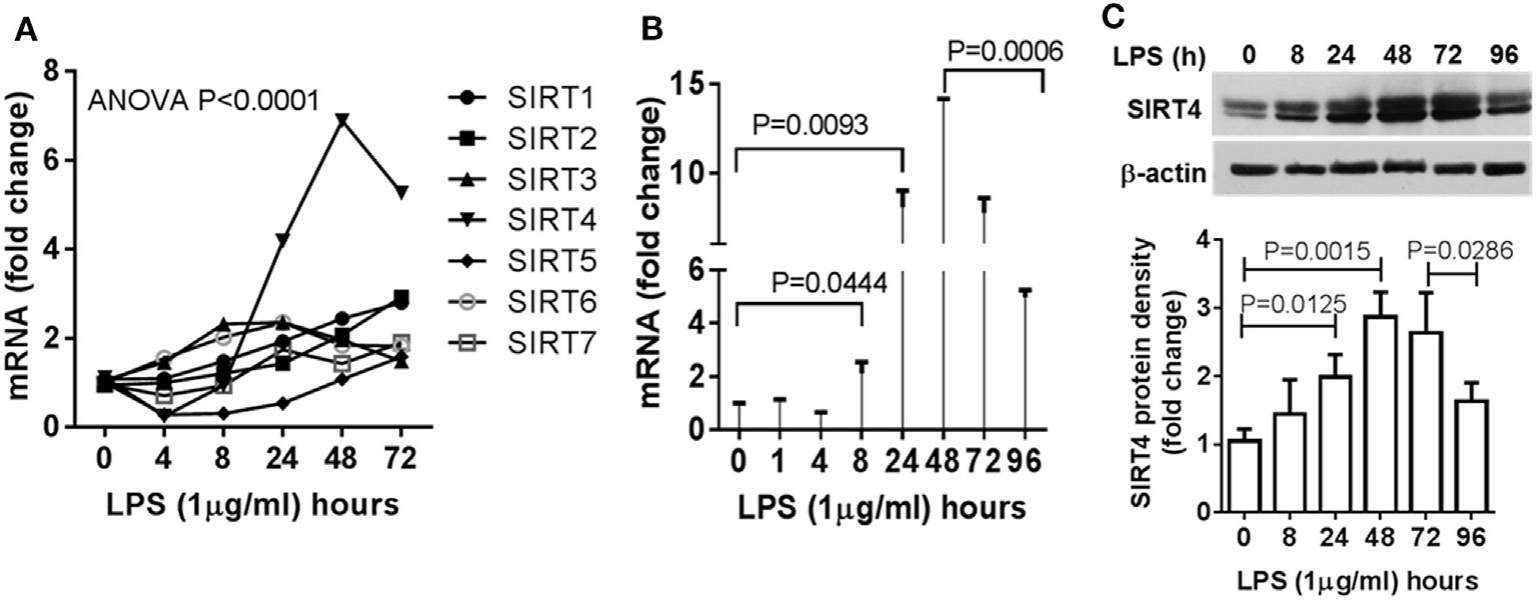
Dynamics of sirtuin 4 (SIRT4) induction during early resolution of the acute inflammation response. THP-1 cells were stimulated with 1 µg/ml lipopolysaccharide (LPS) for indicated times. Gene expression dynamics of mammalian sirtuin family members **(A)** and SIRT4 gene transcription **(B)** were evaluated using real-time RT-PCR analysis. **(C)** Changes of SIRT4 protein level were determined by immunoblotting. Triplicate mean values ± SE of one of five independent experiments displays in bar graph **(A**–**C)**, *N* = 3. *P* values from analysis of variance and *post hoc* comparisons.

Since mitochondrial SIRT4 may counter the effects of SIRT3 (14), which we reported links to SIRT1 epigenetic reprogramming of immune tolerance (8), we hypothesized that SIRT4 activity might contribute to acute inflammatory resolution by countering SIRT1 and SIRT3 effects support of catabolic immune repressor pathways. To test this possibility, we determined the dynamics of gene expression of the seven mammalian sirtuin members in THP-1 cell model of acute inflammatory response. In contrast to the moderate increases in SIRT1, SIRT3, and SIRT6 gene expression during the early phase, SIRT4 was the only gene that was dramatically induced during the tolerance phase (Figure 2A). We further detailed the time course of SIRT4 expression and found that LPS stimulation did not significantly induce SIRT4 expression at 8 h (*P* = 0.0666), but its mRNA level was significantly increased at 24 h (*P* = 0.0093), reached its peak at 48 h, and then returned toward baseline (*P* = 0.0006, Figure 2B). Consistent with an increase in gene expression, we found elevated SIRT4 protein levels at 24 h, with peaking at 48 h (Figure 2C). The delay in SIRT4 induction by LPS paralleled with the time course of endotoxin tolerance emergence, suggesting SIRT4 might play a role in counteracting effects of SIRT1-RelB-SIRT3 immune tolerance axis during recovery of acute inflammatory response.

### SIRT4 Regulates Glycolysis

**Figure 3:**
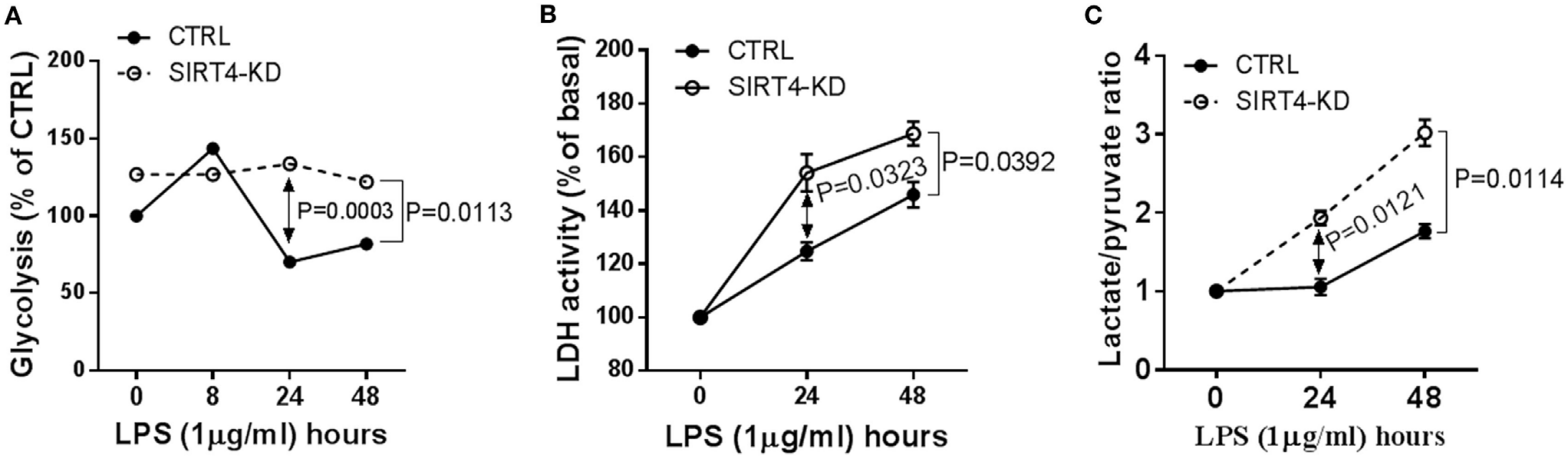
Depleting sirtuin 4 (SIRT4) sustains glycolytic activity during physiological resolution of acute inflammatory response. Control knockdown and SIRT4 knockdown THP-1 cells were stimulated with 1 µg/ml lipopolysaccharide (LPS) for indicated times. **(A)** Cellular glycolytic activity was assessed by the conversion of d-[5-^3^H(N)]glucose to ^3^H_2_O, the changes of ^3^H_2_O generation rate in control and SIRT4 knockdown cells were plotted. **(B)** Changes of lactate dehydrogenase (LDH) activity and **(C)** Changes of ratio of cellular lactate to pyruvate level in both control and SIRT4 knockdown cells were assayed using colorimetric assay kit. One of three independent experiments is presented as mean values ± SE of triplicates. KD, knockdown.

Since glucose is a substrate for glycolysis and contributes to immune anabolic resistance mechanisms, which are repressed by the SIRT1-SIRT6 axis while increasing fatty acid as substrate for catabolic energetics (10), we tested whether SIRT4 participates in the regulation of glycolysis dynamics during inflammatory resolution. Using gene-specific RNAi technology, we assessed glycolysis dynamics by monitoring ^3^H_2_O production of ^3^H-glucose in THP-1 cells. In response to LPS stimulation, ^3^H_2_O generation in control THP-1 cells increased at 8 h, decreased at 24 h and returned toward baseline at 48 h. In contrast, SIRT4 knockdown sustained high ^3^H_2_O count at 24 (*P* = 0.0003) and 48 h (*P* = 0.0113) compared with control vector effects (Figure 3A). Consistent with radiolabeling results, biochemical analysis showed significant increases in LDH activity (Figure 3B) and increased the lactate to pyruvate ratio (Figure 3C) in SIRT4 knockdown cells comparing to that of control THP-1 cells at 24 and 48 h after LPS stimulation. These data suggested that SIRT4 enhances glycolysis-dependent increases in pyruvate support of glucose oxidation, and a possible reversal mechanism for immune tolerance (10).

### SIRT4 Increases PDC Activity by Repressing PDK1 Expression

**Figure 4:**
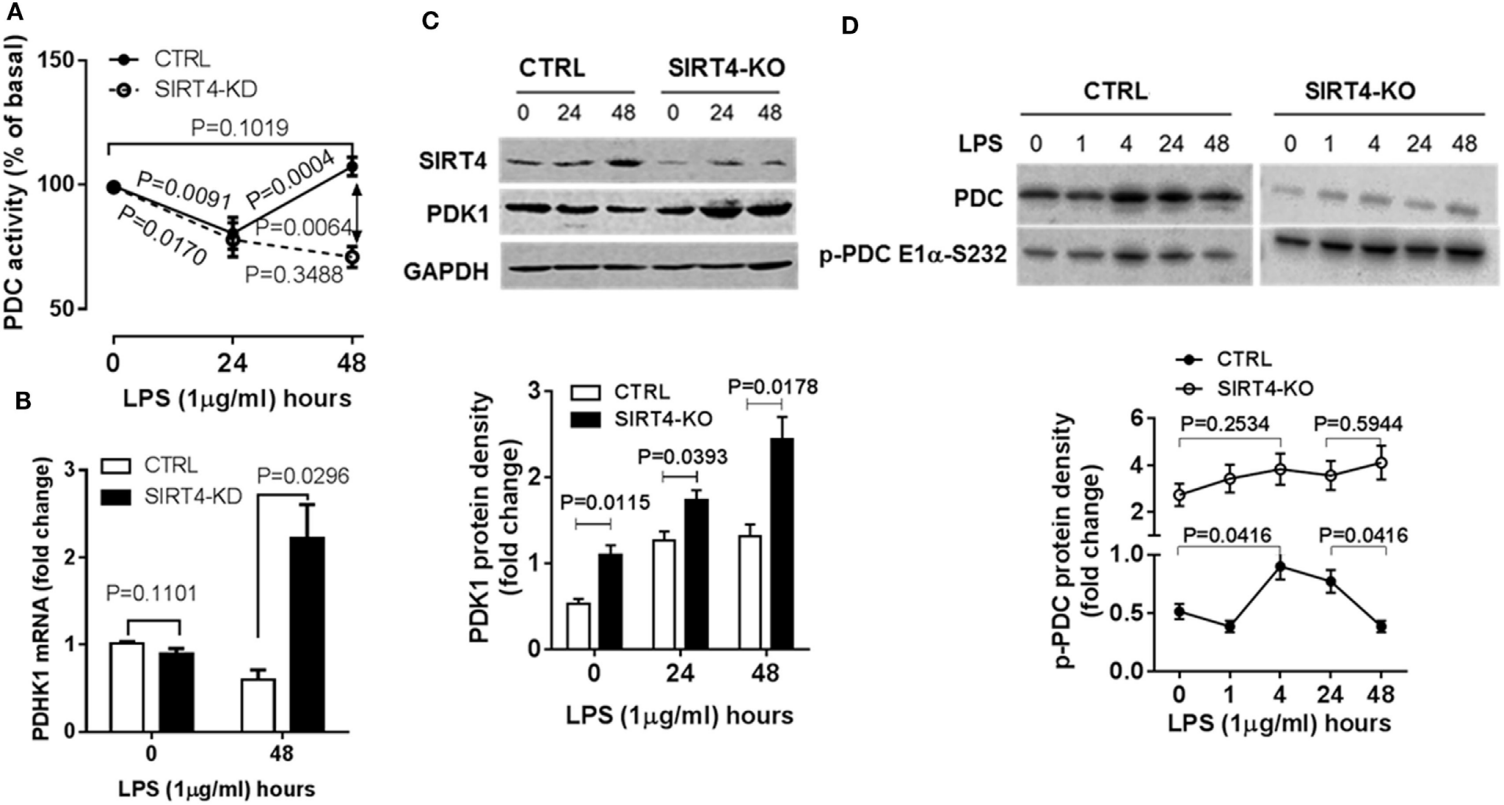
Depleting sirtuin 4 (SIRT4) hampers recovery of PDC inhibition by increasing PDK1 expression during physiological resolution of acute inflammatory response. Control and SIRT4 knockdown or control and SIRT4 knockout THP-1 cells were stimulated with 1 µg/ml lipopolysaccharide (LPS). **(A)** PDC decarboxylase activity was evaluated by commercial colorimetric assay kit (*N* = 3). **(B)** PDK1 mRNA transcription was measured using real-time RT-PCR. PDK1 protein levels **(C)** and PDC protein levels and S232 phosphorylated PDC E1α levels **(D)** were shown by immunoblotting. Bar graphs depict mean values ± SE of *N* = 3. PDC, pyruvate dehydrogenase complex, PDK1, pyruvate dehydrogenase kinase 1, GAPDH, glyceraldehyde 3-phosphate dehydrogenase, KD, knockdown, KO, knockout.

The decarboxylase activity of PDC by increasing acetyl CoA levels in mitochondria directs the oxidation of glucose-based carbons by the TCA cycle and in doing so may alter the cytosol glycolysis to glucose oxidation ratio. To study the dynamics of PDC activity, we used a biochemical assay of PDC enzymatic activity in wild-type and SIRT4 knockdown THP-1 cells. In response to LPS stimulation, PDC activity significantly decreased at 24 hour in both control and SIRT4 deficient cells (*P* = 0.0091, *P* = 0.0171, respectively). PDC enzymatic activity of control cells significantly increased at 48 h (*P* = 0.0004) and restored to the basal level (*P* = 0.1019). In contrast, PDC enzymatic activity in SIRT4 knockdown cells did not change significantly at 48 h compared to 24 h (*P* = 0.3488), but was significantly lower than that of control cells (*P* = 0.0064, Figure 4A). These findings suggest that SIRT4 expression restores PDC enzymatic activity, the major rate-limiting step in glucose oxidation, as immune tolerance resolves and catabolic energetics switch toward glucose driven anabolic oxidative fueling.

Pyruvate dehydrogenase complex activity is primarily regulated by PDK-mediated posttranslational phosphorylation of its E1α subcomponent on specific serines (10, 18). We asked if SIRT4 expression alters PDK1 regulation of PDC during acute inflammatory resolution by comparing PDK1 gene expression in control and SIRT4 knockdown THP-1 cells in response to LPS stimulation. After stimulating for 48 h, the peak time of SIRT4 induction, control THP-1 cells decreased PDK1 mRNA levels. In contrast, SIRT4 knockdown significantly increased PDK1 mRNA level (*P* = 0.00296, Figure 4B).

To determine if PDK1 transcription was efficiently translated, we established SIRT4 knockout THP-1 cells using CRISPR/Cas9 strategy. Consistent with the dynamics of SIRT4 and PDK1 mRNA levels, LPS stimulation induced a time-dependent increase in SIRT4 protein levels while decreasing PDK1 protein levels in wild-type THP-1 cells. In contrast, PDK1 protein level gradually increased in SIRT4 knockout cells (Figure 4C).

We then tested the association of PDK1 induction with PDC E1α phosphorylation. LPS stimulation dramatically induced PDK1 phosphorylation at residue functional serine 232 (S232) on E1α at 1 h, sustained the high level at 24 h, at which time it decreased to basal level at 48 h in wild-type THP-1 cells. In contrast, SIRT4 knockout increased the basal level of phosphorylated PDC E1α-S232 and maintained high levels in response to LPS stimulation (Figure 4D). This paralleled low level of PDC activity in SIRT4 knockdown THP-1 cells (Figure 4A).

### SIRT4 Alters Mitochondrial Respiration

**Figure 5:**
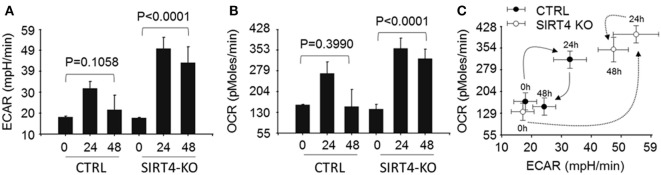
Depleting sirtuin 4 (SIRT4) impedes restoration of energetic homeostasis during physiologic resolution of acute inflammatory response. Control knockout and SIRT4 knockout THP-1 cells were stimulated with 1 µg/ml lipopolysaccharide (LPS) for 0, 24, or 48 h, changes of energetics were examined using Seahorse XFe-24 analyzer, the difference of basal oxygen consumption rate (OCR) **(A)** and extracellular acidification rate (ECAR) **(B)** in response to LPS stimulation in the presence or absence of SIRT4 was presented as mean values ± SE of three independent experiments in **(A,B)**. OCR to ECAR ratio **(C)** is plotted as mean values ± SE of multiple reading points within 8 min measurements, *N* = 3.

We next asked the effect of SIRT4 on glucose oxidation by PDC activation might change mitochondrial respiration. To test this, wild-type and SIRT4 knockout THP-1 cells were stimulated with LPS across time points that estimate basal, resistant, and tolerant states (0, 24, and 48 h) and measured changes of OCR and ECAR using the Agilent Seahorse respiration analyzer. OCR and ECAR were significantly increased at 24 h and returned to baseline at 48 h (*P* = 0.1058) in control knockout cells stimulated with LPS. In contrast, OCR and ECAR were dramatically enhanced at 24 h and sustained high levels for 48 h in SIRT4 knockout cells following LPS stimulation (Figures 5A,5B; Figure S1 in Supplementary Material). We assessed the cell energy index as OCR/ECAR, and found that it returned to baseline at 48 h in control knockdown cells, but remained high in SIRT4 knockdown cells (Figure 5C; Figure S1 in Supplementary Material). These data suggest that SIRT4 rebalances mitochondrial bioenergetics by increasing PDC activation as immune tolerance decreases and inflammation resolves toward a homeostasis state.

### SIRT4 Represses SIRT1 Expression during the Monocyte Acute Inflammatory Response

**Figure 6:**
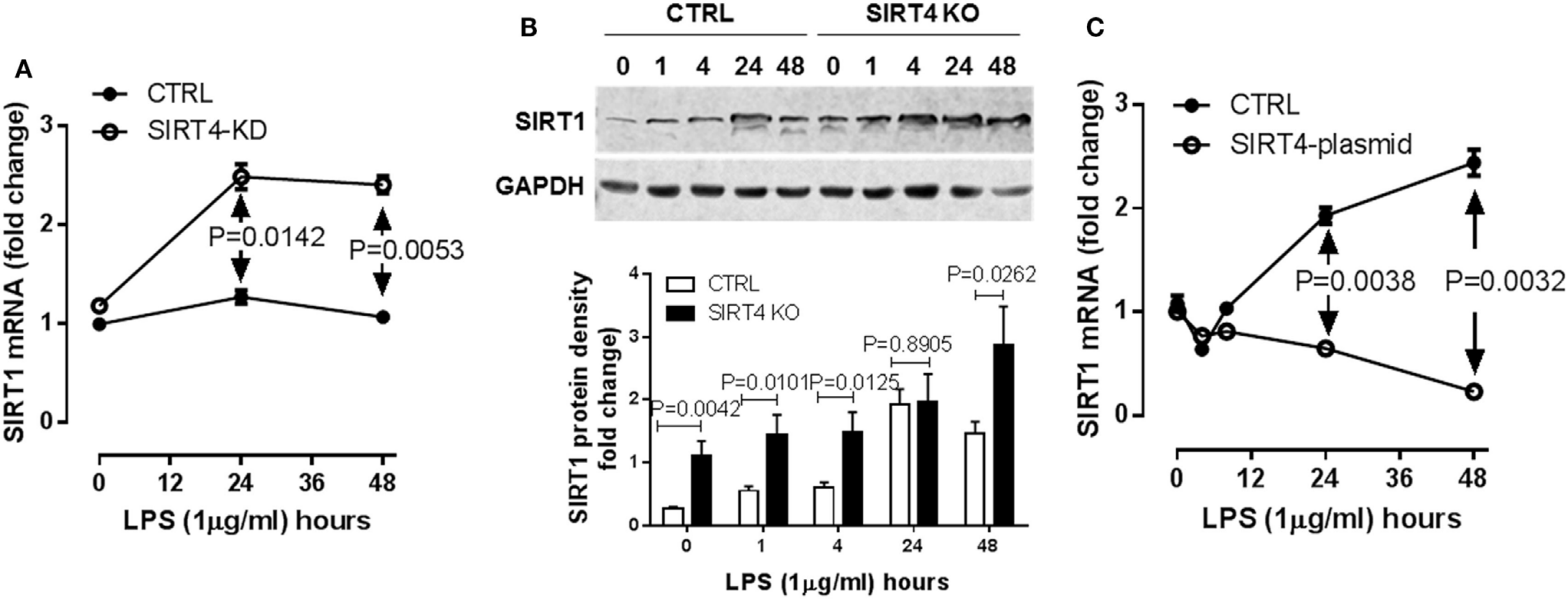
Sirtuin 4 (SIRT4) represses SIRT1 expression in response to endotoxin. Control knockdown and SIRT4 knockdown THP-1 cells were stimulated with 1 µg/ml lipopolysaccharide endotoxin (LPS) for indicated times, **(A)** SIRT1 transcription was evaluated using real-time RT-PCR, data are shown as mean values ± SE of one of two independent experiments. **(B)** Changes of SIRT1 proteins in control and SIRT4 knockout cells were shown by immunoblotting. **(C)** THP-1 cells were electronically transfected either with SIRT4 plasmid or control plasmid for 24 h and stimulated with 1 µg/ml LPS for indicated times, SIRT1 transcription was evaluated using real-time RT-PCR, data are displayed as triplicate mean values ± SE of one of *N* = 3. Bar graphs depict mean values ± SE of *N* = 3.

SIRT1 is master metabolic sensor and regulator and key energy rheostat during the acute inflammatory response, and drives immune resistance to immune tolerance (8). Blocking SIRT1 during the immune tolerance sepsis phenotype in mice restores immunometabolic and energy homeostasis, resolves systemic inflammation and markedly improves survival (19). It also may epigenetically regulate a set of genes that includes PDK (10). We reasoned SIRT4 might counteract SIRT1 activity to promote physiological resolution of acute immunometabolic response. To test this possibility, we assessed LPS-mediated SIRT1 gene expression in the SIRT4 knockdown or SIRT4 overexpressing cells. In response to LPS stimulation, SIRT4 knockout cells expressed significantly higher SIRT1 mRNA than that of control knockdown cells at 24 (*P* = 0.0142) and at 48 h (*P* = 0.0053, Figure 6A). In support of this observation, SIRT1 protein level was increased in SIRT4 knockdown cells comparing to that of control knockdown cells (Figure 6B). Moreover, overexpression of SIRT4 significantly suppressed SIRT1 transcription at 24 h (*P* = 0.0038) and at 48 h (*P* = 0.0032) (Figure 6C). Thus, SIRT4 may contribute to physiological resolution of acute inflammation by limiting SIRT1 control over specific gene sets during immune tolerance in monocytes.

### SIRT4 Reprograms Immune Tolerance in Human Peripheral Blood Mononuclear Cells

**Figure 7:**
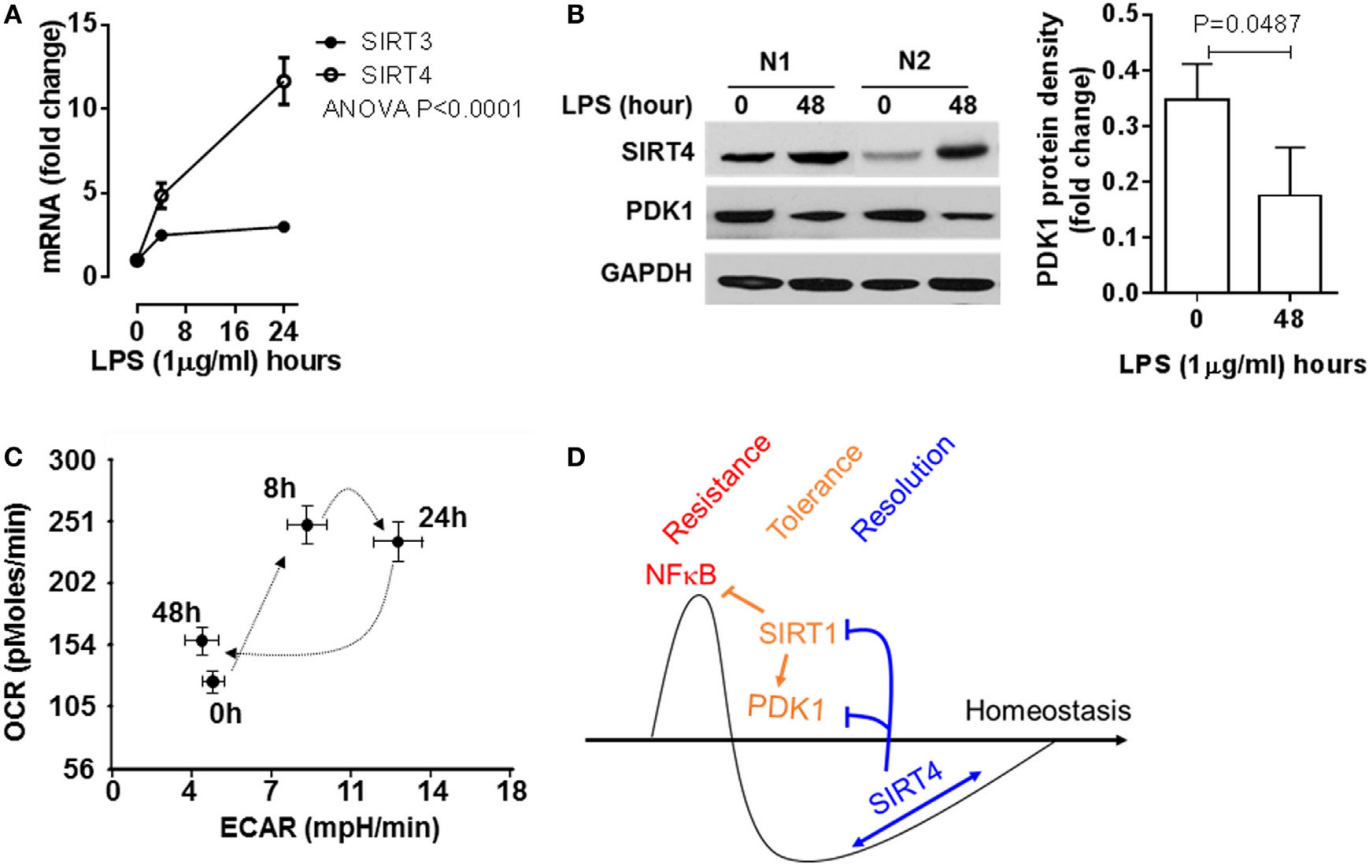
Sirtuin 4 (SIRT4) expression and the mitochondrial bioenergy indices are reprogrammed in human primary blood mononuclear cells during an acute inflammatory response. Healthy peripheral blood mononuclear cells were stimulated with 100 ng/ml lipopolysaccharide (LPS) for indicated times, **(A)** SIRT3 and SIRT4 gene induction was assessed using real-time-RT-PCR analysis (*n* = 3). **(B)** Protein levels of SIRT4 and pyruvate dehydrogenase kinase 1 (PDK1) were detected by western blotting. **(C)** Cellular energetic changes of OCR to extracellular acidification rate (ECAR) ratio were evaluated using Seahorse XFe-24 analyzer (*N* = 3). **(D)** Schematic of mechanism of SIRT4 regulating inflammatory resolution. *P* values from analysis of variance and *post hoc* comparisons. Bar graphs depict mean values ± SE of *N* = 5.

To add support of concept in THP-1 cells, we used human primary blood mononuclear cells stimulated with high dose LPS (100 ng/ml), and monitored SIRT4 and PDK1 expression and mitochondrial energy indices by the Seahorse mitochondrial stress test. In principle with our observations in THP-1 cells, LPS stimulation significantly induced SIRT4 gene transcription (Figure 7A) and protein translation (Figure 7B). PDK1 protein levels decreased when SIRT4 levels increased, suggesting a bioenergy switch (Figure 7B). In support of this, OCR/ECAR ratio energy increased at 8 h to reflect an anabolic fueling, sustained at 24 h and returned toward baseline at 48 h (Figure 7C; Figure S2 in Supplementary Material). Together, these data support our THP-1 derived concept in a primary immune cell circulating population strongly stressed over time by endotoxin.

## Discussion

This study shows that NAD-dependent SIRT4 is a physiologic contributor to acute inflammatory resolution in human monocytes that acts by breaking immune tolerance. The first step in this reprogramming pathway is that SIRT4 expression is selectively increased over other SIRTs as immune tolerance peaks and TNF α expression capacity returns. SIRT4 increases the ratio of glucose oxidation to fatty acid oxidation, as previously reported after inhibiting SIRT1 (9). This study shows SIRT4 physiologically converts the FAO pathway for catabolic energetics to glucose oxidation. As a mechanism by which increased SIRT4 controls energy use and distribution, this study shows that SIRT4 acts by controlling the expression of PDK1 and SIRT1 (Figure 7D), which are master metabolic bioenergy sensors and metabolic regulators of cell energetics and metabolism, as found in tolerant monocytes, septic mice splenocytes, and septic human blood monocytes (10). The clinical importance of this metabolic and immune switch by SIRT1-PDC/PDK1 axis is that blocking SIRT1 in septic mice resolves immune and metabolic tolerance and improves survival in septic mice (19). This study also highlights the importance of substrate selection and bioenergetics in driving innate immune cell fate and function (5–7). This substrate selection paradigm of glucose and amino acids for immune resistance and FAO for immune tolerance applies to innate and antigen specific cells (5–7).

In this study, mitochondrial SIRT4 appears to be indirectly promoting anabolism by increasing glucose metabolism after inhibiting expression of mitochondrial PDK1, which increases glycolysis and opens the pyruvate gate for glucose oxidative fueling. In contrast, mitochondrial SIRT3 directly supports catabolic energy by supporting the functions of TCA cycle proteins (20) and by activating glutamate dehydrogenase (21), which SIRT4 can counter (22). Mitochondrial SIRT4 further supports anabolic biosynthetic processes by repressing malonyl-CoA decarboxylase to increase lipid biosynthesis (23). In contrast, SIRT 3 promotes long-chain fatty acid β oxidation (24) as catabolic fuel during states of nutrient deficiency, which is typical of tolerance. Whether or how SIRT4 and SIRT3 directly control PDC function is unclear. SIRT4 is reported to directly inactivate rather activate PDC (25), but these effects as a lipoamidase and deacylase are controversial (26–28).

A surprising finding in this study is that SIRT4 may signal nuclear processes from its known mitochondrial site, although that SIRT4 may transfer into the nucleus is not excluded in this study. However, there are precedents for how mitochondrial SIRT4 may cross signal to the nucleus. One way is by generating reactive oxygen species and converting superoxide to H_2_O_2_ as a diffusible signaling molecule (29). For example, mitochondrial generated H_2_O_2_ activates the cytoplasmic Keap-1/Nrf2 antioxidant system (30). H_2_O_2_ also directly and reversibly oxidizes nuclear SIRT1 (31) and SIRT6 cysteine thiols (32) to inactivate or reactivate glycolysis and glucose transport during the acute inflammatory response in monocytes. Other mitochondrial signaling pathways could link SIRT4 mitochondrial effects with the nucleus, including levels of citrate (33), fumarate (34), succinate (35), and alpha ketoglutarate (36). Other important unanswered questions raised by this study are: what nuclear transcription and epigenetic system supports SIRT4 transcription and translation and movement to mitochondria, and how SIRT1 and PDK1 and SIRT4 are reciprocally controlled by transcriptional and epigenetic processes.

In summary, we show that SIRT4 operates at the intersection of inflammation tolerance and resolution in monocytes. It reverses fatty acid energy expenditure to glucose energetics by controlling expression and functions of the nuclear SIRT1 axis and the mitochondrial PDC/PDK1 axis. These two cross-roads restore lipid biogenesis as one pathway that retrieves the biosynthetic needs of immunometabolic homeostasis. Expanding this cell-based concept at the mechanistic level and translating it to human or animal diseases may provide clarity to understanding and treating inflammatory diseases.

## Ethics Statement

The Shanghai Public Health Clinical Center Ethics Committee of Fudan University approved the human subject component of this study and the consent form.

## Author Contributions

Conception/design of study: TL and CM. Acquisition of data: TL, JT, JZ and YL. Analysis/interpretation of data: TL, CM, and YL. Drafting the manuscript: TL and CM. Approval of manuscript: TL and CM.

## Conflict of Interest Statement

The authors declare that the research was conducted in the absence of any commercial or financial relationships that could be construed as a potential conflict of interest.
