# Supplementary material for: Mitochondrial Sirtuin 4 Resolves Immune Tolerance in Monocytes by Rebalancing Glycolysis and Glucose Oxidation Homeostasis
Source: Front Immunol. 2018 Mar 9;9:419. doi: 10.3389/fimmu.2018.00419 (PMC5854658; doi:10.3389/fimmu.2018.00419)
Supplement: Supplementary file 1 [file presentation_1.PDF]

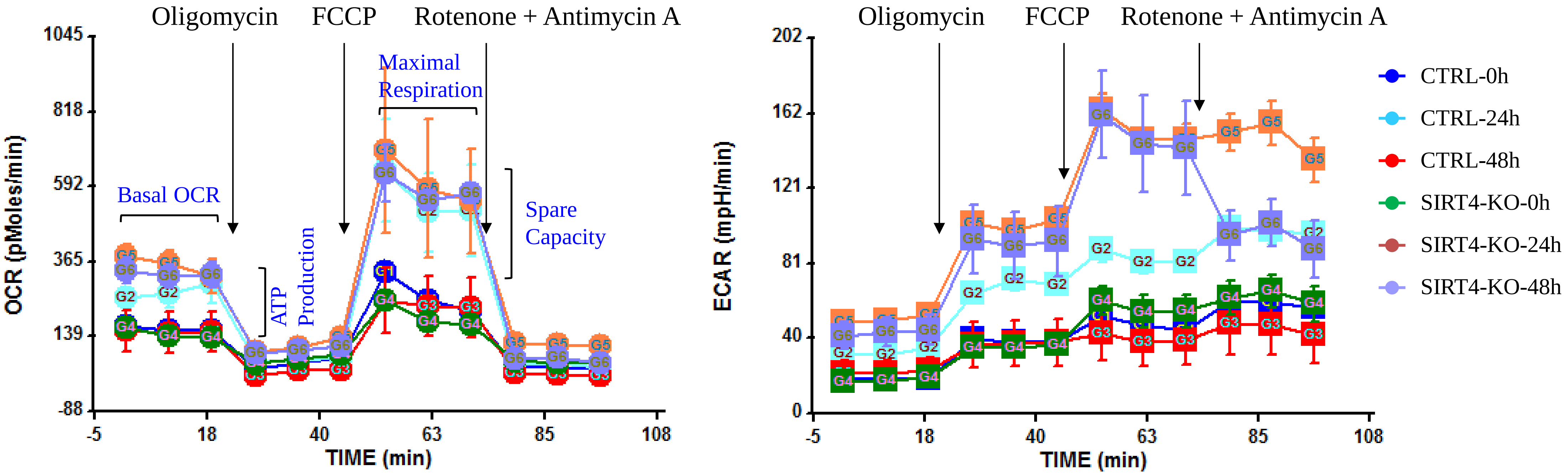

Supplemental Figure 1: Depleting SIRT4 impedes restoration of energetic homeostasis during physiologic resolution of acute inflammatory response. Control knockout and SIRT4 knockout THP-1 cells were stimulated with 1 μg/ml LPS for 0, 24 or 48 hours, changes of energetics were examined using Seahorse XFe-24 analyzer, the difference of OCR (A) and ECAR in response to LPS stimulation in the presence or absence of SIRT4 was presented as mean values ± S.E. of multiple reading points within 8 minute measurements from one of three independent experiments.

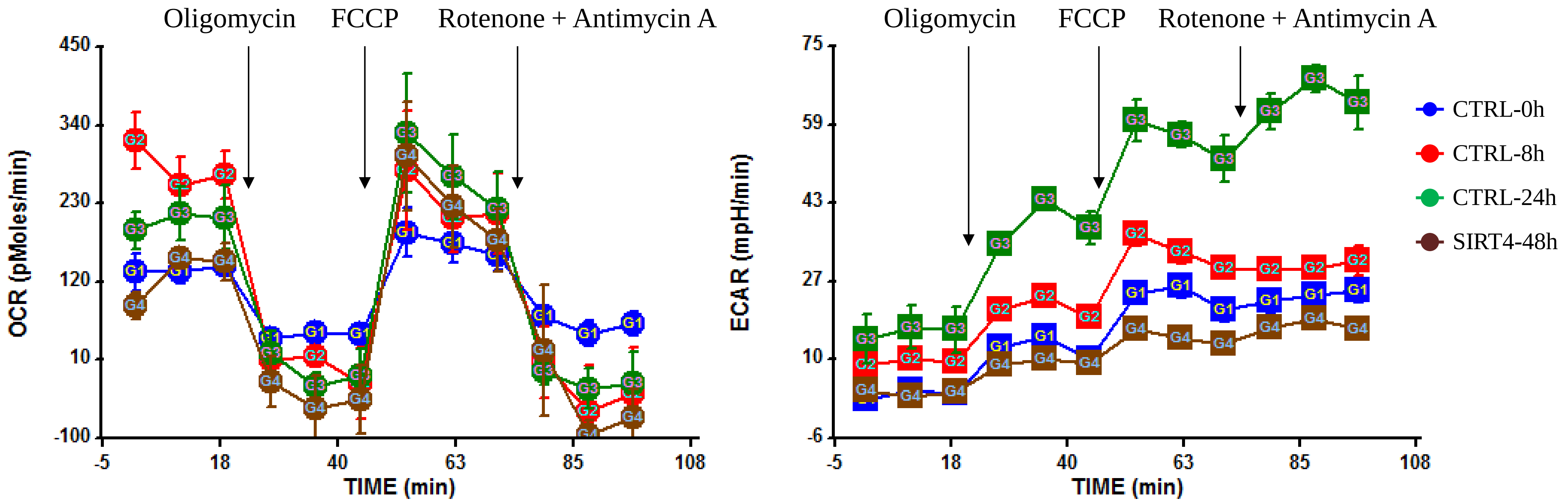

Supplemental Figure 2: Mitochondrial bioenergy indices are reprogrammed in human PBMCs during an Acute Inflammatory Response. Healthy peripheral blood mononuclear cells were stimulated with 100 ng/ml LPS for 0, 8, 24, and 48 hours, Cellular energetic changes of OCR and ECAR were evaluated using Seahorse XFe-24 analyzer, data was presented as mean values ± S.E. of multiple reading points within 8 minute measurements from one of three independent experiments.
